# Correction: ECP-IEM: Enhancing seasonal crop productivity with deep integrated models

**DOI:** 10.1371/journal.pone.0325815

**Published:** 2025-05-29

**Authors:** Ghulam Mustafa, Muhammad Ali Moazzam, Asif Nawaz, Tariq Ali, Deema Mohammed Alsekait, Ahmed Saleh Alattas, Diaa Salama AbdElminaam

The first author, Ghulam Mustafa, is incorrectly noted as the corresponding author. The correct corresponding author is Deema Mohammed Alsekait. Deema Mohammed Alsekait’s email address is: dmalsekait@pnu.edu.sa.
