# Supplementary material for: ECP-IEM: Enhancing seasonal crop productivity with deep integrated models
Source: PLoS One. 2025 Feb 5;20(2):e0316682. doi: 10.1371/journal.pone.0316682 (PMC11798460; doi:10.1371/journal.pone.0316682)
Supplement: Attachment — Submitted filename: Comment_PONE-D-24-34725.pdf [file pone.0316682.s001.pdf]

Reviewer's Comments:

1. **Strengths:**
   - The manuscript demonstrates a clear and well-structured research approach with a novel integration of Bi-GRU and Time Series CNN for crop yield prediction.
   - The study employs rigorous data preprocessing, feature selection, and validation techniques, ensuring robustness and reliability in its findings.
   - The results are promising and indicate significant improvements over existing models, making the research valuable for the agricultural community.
2. **Areas for Improvement:**
   - **Data Accessibility**: To enhance reproducibility and transparency, the authors should provide direct links or clear guidance on accessing the datasets used.
   - **Literature Review Depth**: While the manuscript does include a literature review, it could benefit from a deeper discussion on how the proposed model compares to recent advancements in the field, including potential limitations and areas for future research.
   - **Clarity in Presentation**: Some sections, particularly those involving complex mathematical equations and algorithm descriptions, could be further clarified to enhance readability for a broader audience.
3. **Recommendation:**
   - **Revise and Resubmit**: The manuscript is well-structured, methodologically sound, and presents a novel approach with significant potential impact. However, minor revisions are needed to improve data accessibility, deepen the literature review, and enhance clarity in certain sections. Once these improvements are made, the manuscript will be suitable for publication in PLOS ONE.
