# Supplementary material for: ECP-IEM: Enhancing seasonal crop productivity with deep integrated models
Source: PLoS One. 2025 Feb 5;20(2):e0316682. doi: 10.1371/journal.pone.0316682 (PMC11798460; doi:10.1371/journal.pone.0316682)
Supplement: Attachment — Submitted filename: Reviewer Comments CROP.docx [file pone.0316682.s002.docx]

**Response to Editor’s Comments**

**Title: ECP-IEM: Enhancing Seasonal Crop Productivity with Deep Integrated Models**

We are very thankful to the editor for his decision and reviewers for their valuable suggestions. We have revised the whole manuscript, incorporated all the comments, and highlighted the changes in the revised draft. We hope that the revised manuscript is now according to the required standard. The response to the reviews is summarized as below:

| **S. No.** | **Reviewers Comments** | **Author Reply:** |
| --- | --- | --- |
| **Editor Comments** | | |
| 1 | The novelty of the research is incremental. The authors have combined existing approaches, without clear justification for the choice of the underlying algorithms, and proposed the use of an ensemble approach, i.e., CNN and Bi-GRU. The concern here is a) why these approaches were selected,  b) what were the various other approaches that were considered,  c) what was the methodology, and experimental results to support the choice, pursued to select the two methods  d) using two underlying systems, one for each modality, does not constitute an ensemble. | Thank you for the comments   a. CNN was chosen for its strength in extracting spatial features from satellite imagery and environmental maps, while Bi-GRU was selected for its ability to handle temporal sequences, such as weather and crop yield data, capturing both past and future dependencies. Together, they address both spatial and temporal aspects crucial for accurate crop yield prediction. b. Other approaches considered include LSTM for time-series data, and Random Forest and Gradient Boosting for general prediction tasks. However, CNN and Bi-GRU outperformed them in terms of accuracy and computational efficiency when handling the diverse data in this study. c. The models were chosen after testing multiple configurations, including LSTM and Random Forest, through cross-validation and hyperparameter tuning. CNN-Bi-GRU consistently performed better across accuracy, precision, and recall on all datasets. d. The manuscript has been updated and the word “ensemble” has been removed. |
| 2 | The research contributions of the work are not related to the novelty and performance, but rather they relate to the impact of the application area, which is indeed common with all other works in this field. | The research contributions have been updated. |
| 3 | The authors should provide a detailed description of the datasets, and the specific challenges associated with each dataset. This will allow them to justify the choice of architectures/methods. | A detailed description of the datasets has been added to the manuscript, highlighting their specific characteristics and the challenges associated with each. This includes factors such as the temporal and spatial dimensions of the data, missing values, variability in environmental conditions and imbalanced classes. The updated section provides clarity on how the chosen methods effectively tackle these issues. |
| 4 | The ensemble approach is typically based on using simpler models, which provide for complementary features and decisions, and is characterized by a larger number of models. The larger number of models offers robustness and complementarity demonstrating better performance than that of the underlying models. In this work, there is fusion of two systems, rather than an ensemble, however, there is no substantial evidence (in terms of results) that this leads to better performance than each of the two systems. The recommendation is to consider multiple approaches to extract information from the original image and time series data, which can be combined together into a robust group of features. | The recommendation to explore multiple approaches for extracting information from the original image and time series data, combining them to form a robust feature set, has been duly incorporated. |
| 5 | It is not clear what the specific architectures for the CNN and the Bi-GRU are. What was the process for designing these architectures? The authors should provide information on the design process, including experimental results to support their final architectural recommendations. Ideally, the individual network design performances should be reported. | We acknowledge the need for clarity regarding the specific architectures of the CNN and Bi-GRU models used in the study. In response, we have added detailed information on the design process for both architectures in the manuscript. The architecture selection was driven by extensive experimentation, including hyperparameter tuning, layer depth adjustments, and cross-validation on the datasets to optimize the performance of each model. |
| 6 | Based on the description of the work, the dataset has been balanced prior to training and testing. This introduces data leakage where information from samples that should be used in validation/testing only, has been used in training, which impacts on the accuracy of the results and indeed, it's generalization. Thus, the experiments need to be repeated and depending on the training strategy, the dataset should be split into training and testing, followed by balancing the training set only, and testing on the uncontaminated testing set or using k-fold cross-validation, where at each instance, only the folds of the training set are balanced, while the fold of the validation set remains intact. | We appreciate the reviewer’s insightful observation regarding the potential data leakage due to balancing the dataset before splitting it into training and testing sets. We acknowledge that this approach could lead to the inadvertent introduction of information from the testing set into the training process, thus impacting the model's ability to generalize.  In response, we have revised our experimental methodology to address this concern. The dataset is now first split into training and testing sets, with only the training set undergoing data balancing. The testing set remains untouched, ensuring that it accurately reflects real-world, imbalanced data during evaluation. Additionally, we have repeated the experiments using k-fold cross-validation, ensuring that balancing is applied solely to the training folds, while the validation fold remains intact. These changes ensure that the results reflect the model's true generalization ability. |
| **Reviewer 1** | | |
| 1 | Reviewer #1: 1. The integration of Bi-GRU and Time Series CNN for predicting crop yield is a creative and technically sound solution that addresses the complexity of the variables influencing agricultural output. The use of advanced ensemble methods improves accuracy and robustness. | We appreciate your positive feedback and are pleased that you find the integration of Bi-GRU and Time Series CNN a creative and technically sound approach. |
| 2 | The use of advanced preprocessing techniques, such as Adaptive Synthetic Sampling and feature engineering using SVM and NGD, demonstrates a strong methodological foundation. | Thank you for recognizing our methodological foundation. We believe these techniques are essential for achieving robust and accurate predictions |
| 3 | While the mathematical formulations are sound, a clearer narrative explaining how these methods improve crop yield prediction for a broader audience would enhance the paper's accessibility. | We have revised the manuscript to include a clearer and more detailed explanation of how each method contributes to improving crop yield prediction, ensuring accessibility for a broader audience |
| 4 | While the proposed model shows high accuracy, the limitations of deep learning methods (e.g., need for large datasets, computational complexity) should be discussed in more detail. | We have expanded the discussion of deep learning limitations, addressing the need for large datasets, computational resources, and potential challenges such as overfitting. This section has been highlighted in the updated manuscript. |
| 5 | 5. The datasets used in the study are briefly mentioned, but more detailed descriptions of the datasets, especially regarding their specific characteristics and challenges, would help readers understand the complexities the model is addressing. | A more detailed dataset description has been added, providing specific information about the datasets' characteristics, challenges, and the preprocessing steps taken to manage these complexities. |
|  | Utilize the following references to improve the introduction section. i. <https://www.doi.org/10.1038/s41598-023-42678-x> ii. Rao, M. V., Sreeraman, Y., Mantena, S. V., Gundu, V., Roja, D., & Vatambeti, R. (2023). Brinjal Crop yield prediction using Shuffled shepherd optimization algorithm based ACNN-OBDLSTM model in Smart Agriculture . Journal of Integrated Science and Technology, 12(1), 710.<https://pubs.thesciencein.org/journal/index.php/jist/article/view/a710> | We have incorporated the suggested references into the introduction to strengthen the background and provide additional context for our work. |
| **Reviewer 2** | | |
| 1 | The manuscript presents a well-defined and organized methodology for improving agricultural production prediction. This is achieved by utilizing an integrated ensemble model that combines Bi-GRU and Time Series CNN. The text provides a complete overview of the phases involved in data collection, preprocessing, feature extraction, and model construction. | We are grateful for the positive feedback on our methodology and have further clarified the systematic approach adopted for preprocessing, feature extraction, and the construction of proposed model. |
| 2 | Approach or systematic procedure The authors include comprehensive information regarding the methodologies utilized, including the use of Support Vector Machine (SVM) for feature selection, Normalized Google Distance (NGD) for correlation analysis, and the Rising Star algorithm for feature ranking. The mathematical expressions and equations underlying the suggested model are clearly articulated, hence improving the clarity and replicability of the study. | The information regarding the use of Support Vector Machine (SVM) for feature selection, Normalized Google Distance (NGD) for correlation analysis, and the Rising Star algorithm for feature ranking has been elaborated. The mathematical formulas have also been clarified to ensure the replicability of the study. |
| 3 | Data Availability: The manuscript utilizes three datasets (ECP-DSI, ECP-DSII, and ECP-DSIII), and provides references to their respective sources. Nevertheless, the publication ought to have direct hyperlinks to these datasets or explicitly indicate where the data may be obtained, so improving data accessibility for future researchers. Data analysis and interpretation: | Direct hyperlinks to the datasets have been added to improve accessibility for future researchers, as suggested. |
| 4 | Ethical Considerations: The research does not present any ethical concerns, and the authors have stated that they have no conflicts of interest. The study excludes the participation of both human and animal subjects, hence reducing ethical problems.  Acknowledgements and financial support Transparency: The authors have explicitly acknowledged the financial support provided by Princess Nourah bint Abdulrahman University, so fulfilling the criteria for transparency in funding and potential conflicts of interest. | We affirm that no ethical concerns are associated with the study, as no human or animal subjects were involved. This has been restated in the updated version. |
| 5 | Originality and Significance: The manuscript introduces a noteworthy breakthrough in the realm of agricultural management and crop production prediction. This is achieved by combining advanced deep learning algorithms with ensemble modeling. The suggested method overcomes the constraints of conventional and current machine learning methods, hence providing more precise and resilient predictions.  Wide-ranging Relevance and Applicability: Crop yield prediction is a widely studied subject, especially due to the worldwide concerns around food security. The discoveries have the capacity to influence agricultural practices by facilitating more effective distribution of resources, control of risks, and development of strategic plans. | Thank you for your insightful and positive feedback on the manuscript. We are delighted to hear that you find the originality and significance of our work noteworthy. Our primary goal was to advance the field of agricultural management through the integration of deep learning algorithms and ensemble modeling, and we are pleased that our proposed method has been recognized for overcoming the limitations of traditional approaches.  We agree that crop yield prediction is indeed a critical topic given global food security concerns. By improving prediction accuracy and robustness, we aim to support more efficient resource allocation, risk management, and strategic planning in agriculture. We are grateful for your appreciation of the broad relevance and potential impact of our findings. |
